# Acupuncture versus cognitive behavioral therapy for pain among cancer survivors with insomnia: an exploratory analysis of a randomized clinical trial

**DOI:** 10.1038/s41523-021-00355-0

**Published:** 2021-11-30

**Authors:** Mingxiao Yang, Kevin T. Liou, Sheila N. Garland, Ting Bao, Tony K. W. Hung, Susan Q. Li, Yuelin Li, Jun J. Mao

**Affiliations:** 1 Department of Medicine, Integrative Medicine Service, Memorial Sloan Kettering Cancer Center, 1429 First Avenue, New York, NY 10021 USA; 2 Department of Psychology and Discipline of Oncology, Memorial University, 232 Elizabeth Avenue, St. John’s, NL A1B 3×9 Canada; 3 Department of Medicine, Division of Solid Tumor Oncology, Head & Neck Oncology Service, Memorial Sloan Kettering Cancer Center, 1275 York Avenue, New York, NY 10065 USA; 4 Department of Psychiatry and Behavioral Sciences, Memorial Sloan Kettering Cancer Center, 1275 York Avenue, New York, NY 10065 USA

**Keywords:** Pain, Outcomes research, Cancer

## Abstract

Pain and insomnia often co-occur and impair the quality of life in cancer survivors. This study evaluated the effect of acupuncture versus cognitive behavioral therapy for insomnia (CBT-I) on pain severity among cancer survivors with comorbid pain and insomnia. Using data from the CHOICE trial that compared acupuncture versus CBT-I for insomnia among cancer survivors, we analyzed the effect of interventions on pain outcomes in 70 patients with moderate to severe baseline pain. Interventions were delivered over eight weeks. We assessed average pain severity (primary outcome) and pain interference at baseline, week 8, and week 20. We further defined insomnia and pain responders as patients who achieved clinically meaningful improvement in insomnia and pain outcomes, respectively, at week 8. We found that compared with baseline, the between-group difference (-1.0, 95% CI -1.8 to -0.2) was statistically significant favoring acupuncture for reduced pain severity at week 8 (-1.4, 95% CI -2.0 to -0.8) relative to CBT-I (-0.4, 95% CI-1.0 to 0.2). Responder analysis showed that 1) with acupuncture, insomnia responders reported significantly greater pain reduction from baseline to week 4, compared with insomnia non-responders (-1.5, 95% CI -2.7 to -0.3); 2) with CBT-I, pain responders reported significantly greater insomnia reduction at week 8, compared with pain non-responders (-4.7, 95% CI -8.7 to -1.0). These findings suggest that among cancer survivors with comorbid pain and insomnia, acupuncture led to rapid pain reductions, which contributed to a decrease in insomnia, whereas CBT-I had a delayed effect on pain, possibly achieved by insomnia improvement.

## Introduction

Pain and insomnia are common and disturbing symptoms that 56–59% of cancer patients and survivors experience^1–5^. Epidemiology studies corroborate the bidirectional relationship between pain and insomnia, where pain affects sleep onset and maintenance, and sleep impairments may further exacerbate pain^6,7^. Pain and insomnia also contribute to the progression of fatigue and mental distress that further worsen symptom burden, leading to poor quality of life^8–10^. In breast cancer patients, pain and psychological symptoms like insomnia and depression are associated with early discontinuation and non-adherence to adjuvant hormonal therapy and, consequently, poor clinical prognosis^11–14^. In recent decades, timely and proper management of insomnia and pain has been identified as a critical element of comprehensive cancer care and several validated therapies have emerged as effective insomnia treatments^15,16^.

Cognitive-behavioral therapy for insomnia (CBT-I) is the “gold standard” nonpharmacologic treatment for insomnia in the general population and in cancer survivors, with medium-to-large effect sizes for sleep outcomes that persist after intervention delivery ends^17–19^. Pain, one of the most common concurrent symptoms of insomnia, is inadequately addressed. Secondary analyses have demonstrated that CBT-I outcomes do not differ for those with and without pain^20^, but the literature is mixed on whether treating insomnia improves pain outcomes in noncancer populations^21,22^. In clinical practice, effective insomnia treatment is expected to result in pain relief due to the putative bidirectional relationship. This effect has been observed as a result of acupuncture for non-malignant pain^23–25^. Acupuncture is a nonpharmacologic modality that is also effective in ameliorating objective and subjective insomnia compared with sham acupuncture^26,27^. The use of acupuncture during cancer treatment and survivorship not only mitigates insomnia but can provide substantial relief of pain and fatigue^28–30^.

In cancer populations, our understandings of the association between pain and insomnia and effective treatments for these comorbidities is limited due to a paucity of research evidence. To inform patient-centered pain management comorbid with insomnia, this secondary analysis focused on: (1) the comparative effectiveness between acupuncture and CBT-I for pain severity (primary outcome) and pain-related interference (secondary outcomes) in cancer survivors with clinically confirmed insomnia diagnoses and moderate to severe baseline pain, (2) exploring whether participants who experienced a clinically meaningful reduction in insomnia after acupuncture or CBT-I (i.e. “responders”) had improvement in pain compared to non-responders, and (3) exploring whether participants who experienced a clinically meaningful reduction in pain after acupuncture or CBT-I had improvement in insomnia compared to non-responders. Such understanding will not only help tailor appropriate treatment for survivors who may experience co-morbid insomnia and pain but also inform the generation of appropriate hypotheses for novel symptom interventions.

## Results

### Demographics and clinical characteristics of participants

**Fig. 1:**
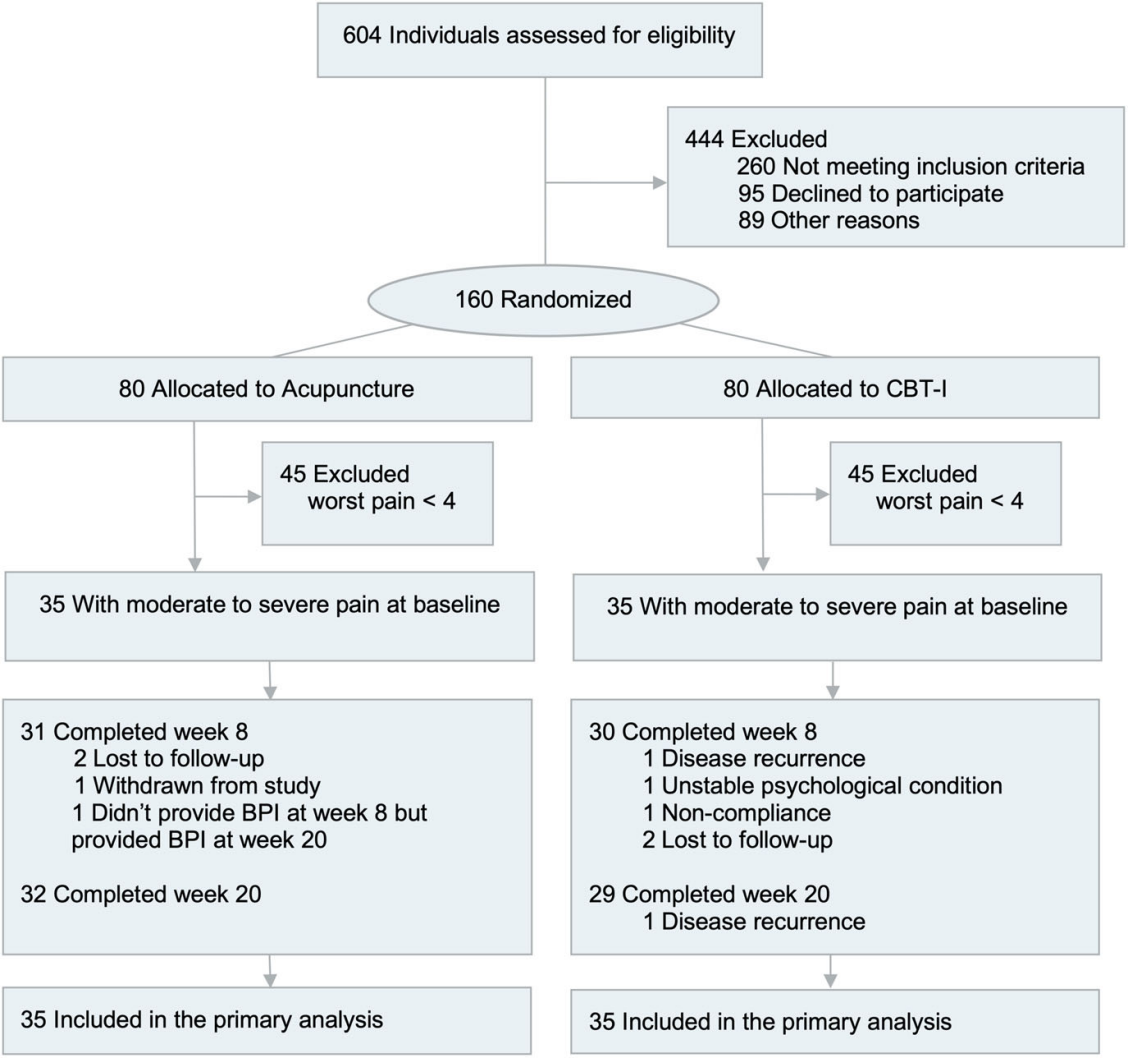
Study flow chart. This study only included participants with comorbid insomnia and pain. Key: CBT-I cognitive behavioral therapy for insomnia, BPI Brief Pain Inventory.

Of the160 participants randomized to receive acupuncture (*n* = 80) or CBT-I (*n* = 80), 35 in each group had a baseline worst pain value of 4 or greater and were included in this analysis (Fig. 1). Of those assigned to acupuncture, 31 completed the required treatment, and 32 completed the 20-week follow-up. Of those assigned to CBT-I, 30 completed the study interventions, and 29 completed the 20-week follow-up. All 70 participants were included in ITT analyses. The demographics and clinical characteristics are detailed in Table 1.

**Table 1:** Demographics and clinical characteristics of participants.

Characteristics	Total (*N* = 70)	Acupuncture (*N* = 35)	CBT-I (*N* = 35)
Age, mean (SD)	61.6 (10.8)	60.8 (11.2)	62.4 (10.5)
*Gender, n (%)*			
Male	27 (38.6)	17 (48.6)	10 (28.6)
Female	43 (61.4)	18 (51.4)	25 (71.4)
*Race, n (%)*			
White	47 (68.1)	27 (77.1)	20 (58.8)
Non-white^a^	22 (31.9)	8 (22.9)	14 (41.2)
*Education, n (%)*			
High school or less	14 (20.0)	9 (25.7)	5 (14.3)
College or above	56 (80.0)	26 (74.3)	30 (85.7)
*Marital status, n (%)*			
Married/living w/partner	35 (50.0)	18 (51.4)	17 (48.6)
Single/divorced/separated/widowed	35 (50.0)	17 (48.6)	18 (51.4)
*Employment, n (%)*			
Full-time	18 (26.1)	14 (41.2)	4 (11.4)
Part-time	11 (15.9)	7 (20.6)	4 (11.4)
Not currently employed	40 (58.0)	13 (38.2)	27 (77.1)
*Cancer type, n (%)*			
Breast	21 (30.0)	8 (22.9)	13 (37.1)
Prostate	12 (17.1)	8 (22.9)	4 (11.4)
Colon/rectal	3 (4.3)	2 (5.7)	1 (2.9)
Head/neck	3 (4.3)	1 (2.9)	2 (5.7)
Hematologic	10 (14.3)	4 (11.4)	6 (17.1)
GYN	4 (5.7)	2 (5.7)	2 (5.7)
Other cancer^b^	10 (14.3)	5 (14.3)	5 (14.3)
More than one cancer	7 (10.0)	5 (14.3)	2 (5.7)
*Cancer treatments*^*c*^*, n (%)*			
Surgery	53 (75.7)	27 (77.1)	26 (74.3)
Chemotherapy	34 (48.6)	17 (48.6)	17 (48.6)
Radiation	37 (52.9)	21 (60)	16 (45.7)
Hormonal	20 (28.6)	10 (28.6)	10 (28.6)
Years since cancer diagnosis, mean (SD)	6.1 (5.7)	6.5 (4.4)	5.7 (6.8)
Years since insomnia onset, mean (SD)	7.5 (6.5)	7.3 (4.9)	7.7 (7.8)
Brief pain inventory severity, mean (SD)	4.2 (1.8)	3.9 (1.6)	4.5 (2.0)
Brief pain inventory worst pain, mean (SD)	6.0 (1.8)	5.6 (1.5)	6.4 (2.1)
Insomnia severity index, mean (SD)	20.3 (36)	19.6 (3.5)	20.9 (3.6)

^a^Majority of the non-white are Black.

^b^Other cancer includes: skin, lung, other GI, other GU, etc.

^c^Subjects can have more than 1 type of cancer treatments.

*CBT-I* cognitive behavioral therapy for insomnia, *SD* standard deviation.

### Effect of acupuncture versus CBT-I on pain severity and pain interference

**Fig. 2:**
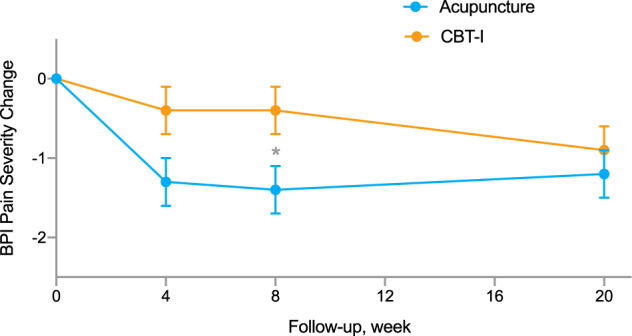
Effect of acupuncture and CBT-I on pain severity. Figure 2 illustrates the trajectory of Brief Pain Inventory severity score changes from baseline to week 20 in the acupuncture versus CBT-I group. The asterisk mark (*) indicates a within-group difference with statistical significance (*p* < 0.05). Error bar indicates standard error of the mean. CBT-I cognitive behavioral therapy for insomnia, BPI Brief Pain Inventory.

From baseline to week 8, acupuncture significantly reduced average pain severity (mean = −1.4 points; 95% confidence interval [CI]: −2.0 to −0.8; *P* < 0.001), whereas CBT-I did not (−0.4 point; 95% CI: −1.0 to 0.2; *P* = 0.21). The between-group difference was statistically significant (−1.0 point; 95% CI: −1.8 to −0.2; *P* = 0.015). From baseline to week 20, significant pain reduction was seen in both acupuncture (−1.2 points, 95% CI: −1.8 to −0.7; *P* < 0.001) and CBT-I (−0.9 point, 95% CI: −1.4 to −0.3; *P* = 0.005) groups; the between-group difference at week 20 was not statistically significant (*P* = 0.37) (Fig. 2, Table 2).

**Table 2:** Change in study outcomes from baseline by treatment.

Characteristics	Change from baseline in acupuncture, mean (95% CI)	Change from baseline in CBT-I, mean (95% CI)	Between group differences, mean (95% CI)	*p*-Value* (between group comparison)
*BPI pain severity*				
Week 8	−1.4 (−2.0 to −0.8)	−0.4 (−1.0 to 0.2)	−1.0 (−1.8 to −0.2)	0.015
Week 20	−1.2 (−1.8 to −0.7)	−0.9 (−1.4 to −0.3)	−0.4 (−1.2 to 0.4)	0.37
*BPI average pain item*				
Week 8	−1.3 (−1.9 to −0.7)	−0.1 (−0.7 to 0.5)	−1.2 (-2.1 to −0.3)	0.0071
Week 20	−1.3 (−1.9 to −0.7)	−0.5 (−1.2 to 0.1)	−0.8 (−1.6 to 0.1)	0.086
BPI worst pain item				
Week 8	−2.1 (−3.0 to −1.2)	−1.0 (−2.0 to −0.1)	−1.1 (−2.4 to 0.3)	0.12
Week 20	−1.5 (−2.4 to −0.6)	−1.3 (−2.3 to −0.4)	−0.2 (−1.5 to 1.2)	0.81
*BPI interference*				
Week 8	−2.1 (−2.8 to −1.5)	−1.5 (−2.2 to −0.8)	−0.6 (−1.6 to 0.4)	0.21
Week 20	−1.5 (−2.2 to −0.8)	−1.9 (−2.7 to −1.2)	0.4 (−0.5 to 1.4)	0.38
*ISI insomnia severity*				
Week 8	−10.3 (−12.1 to −8.6)	−11.4 (−13.2 to −9.6)	1.0 (−1.5 to 3.6)	0.42
Week 20	−10.0 (−11.7 to −8.2)	−12.8 (−14.7 to −11.0)	2.9 (0.3–5.4)	0.028

Note: *p-value was obtained using a linear mixed-effects model.

*CI* confidence interval, *CBT-I* cognitive behavioral therapy for insomnia, *BPI* Brief Pain Inventory, *ISI* Insomnia Severity Index.

Regarding secondary pain outcomes, both interventions significantly improved pain-related interference and worst pain severity from baseline to week 8 and week 20 with no significant between-group differences (Table 2).

### Associations between pain reduction and insomnia improvement during treatment

**Fig. 3:**
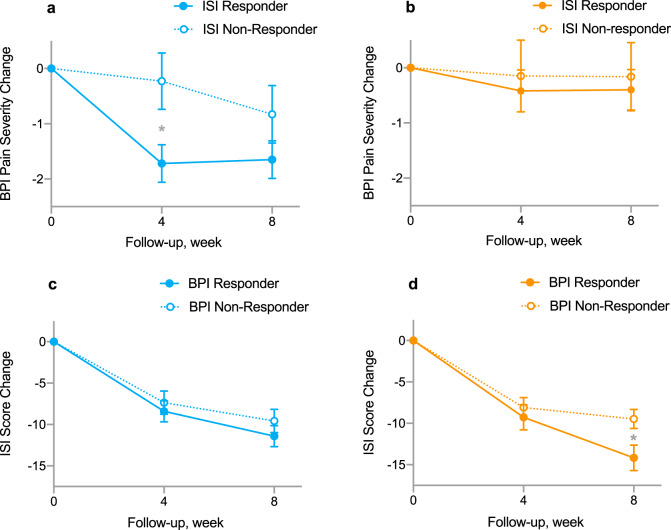
Relationship between pain and insomnia in acupuncture and CBT-I. **a** Patients who responded to acupuncture treatment for insomnia at week 8 was associated with significantly greater pain reduction at week 4 as compared with non-responders; **b** No significant difference of BPI pain severity changes in ISI responder versus non-responder in CBT-I group at either week 4 or 8. **c** No significant difference in ISI score changes between BPI responder and non-responder in acupuncture group at either week 4 or 8; **d** BPI responders in CBT-I group had significantly more reductions in ISI score than BPI non-responders. Error bar indicates standard error of the mean. ISI Insomnia Severity Index, BPI Brief Pain Inventory, CBT-I cognitive behavioral therapy for insomnia.

Compared to baseline, acupuncture resulted in a −10.3-point reduction in the ISI score at week 8 (95% CI: 12.1 to −8.6; *P* < 0.001) compared to a −11.4-point reduction (95% CI: −13.2 to −9.6; *P* < 0.001) in CBT-I. In the acupuncture group, pain reduction at week 4 was greater in insomnia responders than insomnia non-responders (−1.49; 95% CI: −2.7 to −0.3, *P* = 0.016) (Fig. 3a). However, there was no difference between responders and non-responders in the CBT-I group (Fig. 3b). With respect to insomnia outcome, pain responders in the CBT-I group had a greater insomnia reduction at week 8 than non-responders (−4.71; 95% CI: −8.74 to −0.95, *P* = 0.016) (Fig. 3d). However, there was no difference in the acupuncture group (Fig. 3c).

## Discussion

This study is one of the first to compare the effects of acupuncture versus CBT-I in cancer survivors with pain co-occurring with insomnia. Acupuncture demonstrated significant pain reduction at weeks 8 and 20, whereas CBT-I only demonstrated significant pain reduction at week 20. In the acupuncture group, clinically meaningful improvement in insomnia was associated with pain reduction early in the treatment course, suggesting that acupuncture may improve insomnia via pain reduction. In the CBT-I group, clinically meaningful improvement in pain was associated with a reduction in insomnia symptoms, suggesting that CBT-I may improve pain by treating co-occurring insomnia. These findings warrant further investigation to elucidate the differential pathways by which these two interventions may improve pain and insomnia symptoms.

This study increases our understanding of the effect of acupuncture for pain in cancer survivors with insomnia. A growing body of clinical evidence indicates that acupuncture could significantly reduce cancer pain and decrease analgesic use^29^. Our study confirmed these findings by showing a significant, rapid pain reduction in acupuncture treatment relative to CBT-I which contributed to a reduction in insomnia. In a systematic review of patients with both chronic, non-malignant pain and insomnia, acupuncture significantly reduced both symptoms compared with hypnotics^23^. The effects of acupuncture on pain are largely attributed to neurobiological systems/mediators with predominantly analgesic properties (e.g. opioid, monoaminergic, orexinergic, immune, melatonin, and endocannabinoid systems). Many of these pathways overlap with sleep homeostasis^31,32^, thus providing biological plausibility for acupuncture to address both pain and insomnia.

Our study also contributes to the limited understanding of CBT-I for pain in cancer survivors with insomnia. Previous studies in noncancer populations reported that CBT-I was not associated with significant improvements in pain severity at the end of treatment, but there appeared to be a delayed trend at three to 6 months after the intervention delivery^24,25,33^. This is consistent with our findings that CBT-I only produced significant pain reductions three months post-treatment. Prior research has linked sleep deficiency to hyperalgesia^34^. Treatment with CBT-I does not always increase total sleep time in the short term, but in the long term, it is possible that CBT-I may eventually result in pain relief as total sleep time is increased^35^. Consistent with this view, our study found that CBT-I patients with clinically meaningful pain improvements showed a greater reduction in insomnia symptoms compared to patients whose pain did not respond. These findings indicate that insomnia reduction was required to produce meaningful pain improvement among patients receiving CBT-I.

Our findings have clinical meaningfulness for managing pain comorbid with insomnia in cancer populations. For example, among breast cancer survivors using aromatase inhibitors, severe joint pain is independently associated with clinically significant insomnia (adjusted odds ratio 4.84, *P* = 0.003)^36^. A patient-reported worst joint pain score of 4 or greater on the BPI is a significant predictor of premature discontinuation of aromatase inhibitors (hazard ratio 2.09, *P* = 0.016)^37^, leading to increased cancer recurrence and higher mortality rates^12,38^. The more rapid relief of pain achieved by acupuncture (8 weeks vs. 20 weeks) as compared with CBT-I not only provides timely symptom management but may also help survivors adhere to cancer treatment. Future research should evaluate whether the timely assessment of symptoms combined with the provision of acupuncture would lead to better adherence to hormonal treatment for breast cancer survivors.

Our responder analysis provides insight into the bio-behavioral mechanisms of the interventions that can explain the different effects they may have on symptom reduction. The positive association between pain reduction and insomnia response to acupuncture indicates that insomnia improvement achieved by acupuncture is possibly modulated by the activation of pain-related biological pathways^31,32^. However, the association between insomnia improvement and pain response suggests that behavioral modulation of sleep via stimulus control or sleep restriction may reduce pain over time^39–41^. Our observation raises the possibility of whether combining acupuncture with key behavioral components of CBT-I (i.e. stimulus control or sleep restriction) may lead to an intervention that provides both more rapid and sustained improvement in pain and sleep. This hypothesis needs to be further evaluated in prospective trials.

This study has several limitations. First, it is a secondary analysis from an existing trial, which makes our results hypothesis-generating rather than confirmatory. Second, the original CHOICE trial was designed primarily to assess the effect of interventions for insomnia; we do not have detailed clinical information on pain such as pain type (e.g., neuropathic and somatic), duration of pain, or pain medication use. Future research should incorporate detailed phenotypes of pain with objective and subjective sleep measures. Further, since we conducted multiple analyses, there are risks for false discovery; these warrant prospectively designed studies to verify the findings.

Among cancer survivors with comorbid pain and insomnia, acupuncture and CBT-I appeared to improve pain and insomnia through divergent pathways. Acupuncture produced more rapid and sustained reduction in pain severity compared with CBT-I, which contributed to a clinically meaningful insomnia response to acupuncture. In contrast, CBT-I significantly improved insomnia severity, and this insomnia improvement was associated with a delayed, but clinically meaningful pain response to CBT-I. Further research should test whether combining acupuncture with behavioral sleep techniques can lead to greater and more sustained improvements for patients with comorbid pain and insomnia.

## Methods

### Study design

The present study used data from a previously published comparative effectiveness (CHOICE) trial that demonstrated the substantial clinical effect of both acupuncture and CBT-I for insomnia in cancer survivors (ClinicalTrials.gov Identifier NCT02356575, registration date Feb 5, 2015)^30^. The current analyses were embedded as a secondary aim of the published trial. The original study was completed from March 2015 to July 2017. The institutional review boards at the University of Pennsylvania and Memorial Sloan Kettering Cancer Center approved the study procedures summarized below.

### Study participants and procedures

Eligible participants were English-speaking; aged 18 years or above; previously diagnosed with cancer and completed active treatment (surgery, chemotherapy, and/or radiotherapy) at least one month prior to inclusion; scored at least 8 on the Insomnia Severity Index (ISI); and met the diagnostic criteria of insomnia disorder as defined by the Diagnostic and Statistical Manual of Mental disorders, 5th Edition^42^. Exclusion criteria included: (1) a diagnosis of another concurrent sleep disorder not adequately treated; (2) previous experience with or currently receiving CBT or acupuncture to treat insomnia; (3) the presence of a psychiatric disorder not adequately treated; or (4) employment in a job requiring shift work that would impair the ability to establish a regular sleep schedule. Participants using psychotropic medications and/or hypnotics or sedatives were eligible if the dose was stable over the previous 6 weeks. The present study included only participants who scored at least 4 points on the worst pain item of the Brief Pain Inventory (BPI) at baseline.

After initial screening, trained research staff interviewed interested participants to confirm an insomnia diagnosis and additional inclusion/exclusion requirements. Confirmed eligible patients were scheduled to complete baseline sleep assessment after providing written informed consent. Participants were randomly assigned to either the CBT-I or acupuncture group in a 1:1 ratio using a permuted block randomization mechanism. An independent statistician generated the random digit sequence. Study investigators (PI, co-investigators, and statisticians) were masked to group assignment. Patients, research staff, and treatment therapists were not masked.

### Interventions

The acupuncture protocol was a manualized, semi-standardized treatment composed of standardized acupoints to ameliorate insomnia and supplemental acupoints to address comorbid symptoms (such as pain and anxiety) based on individual needs. Patients received acupuncture twice weekly for 2 weeks, followed by weekly treatment for 6 weeks, for a total of 10 treatments over 8 weeks. The first acupuncture visit involved a detailed history and examination lasting 60 min, with each subsequent session lasting 30 min, for a total time of 330 min (total provider contact time was approximately 150 min). The acupuncture treatment protocol and the training of the practitioners are detailed in Supplementary Material.

CBT-I is a manualized, multicomponent treatment that includes sleep hygiene, sleep restriction, stimulus control, cognitive restructuring, and relaxation training^17^. Patients received five weekly sessions of CBT-I followed by two bi-weekly sessions, for a total of seven sessions over eight weeks. The first CBT-I session was 60 min and the remaining sessions were 30 min each, for a total contact time of 240 min. Licensed therapists and doctoral-level psychology trainees delivered CBT-I treatments.

### Outcomes

The BPI is an 11-item pain assessment tool validated for use with cancer patients^43^. It measures the intensity of the pain (four items) and interference (seven items) of pain in the patient’s life. The average score on the four pain severity items is the primary outcome for this analysis. The BPI psychometrics are well-established (Cronbach’s alpha 0.80–0.87 for the 4 pain severity items and 0.89–0.92 for the 7 interference items). Participants who reported a reduction of 33% or greater on average pain intensity after treatment were considered pain responders^44^.

The ISI is a validated patient-reported outcome of insomnia designed to specifically assess the impact of insomnia on daytime functioning and the severity of associated distress^45^. The ISI includes seven items that are scored on a five-point scale ranging from 0 to 4 with higher scores representing more severe insomnia symptoms. The optimal cutoff scores are 0–7 (no clinically significant sleep difficulties), 8–14 (sleep difficulties warrant further investigation), and 15+(presence of clinically significant insomnia). The ISI has demonstrated internal consistency, reliability, construct validity, specificity, and sensitivity^46^. Participants who report a ≥8 reduction in ISI score or achieve an ISI score <8 after treatment are considered insomnia responders^34,47^.

### Statistical analysis

Statistical analyses were performed following intention-to-treat principles. Descriptive statistics were used to report baseline participant characteristics. Changes in outcomes from baseline to weeks 8 and 20 were plotted for the two treatment groups to visualize the patterns of improvement over time. The observed differences were evaluated by linear mixed-effects models with random intercepts to account for the correlation between repeated outcome assessments nested within individual participants. Further, the mixed-effects models fitted the longitudinal outcomes at weeks 0, 4, 8, and 20 as a function of fixed effects of treatment (acupuncture vs. CBT-I), time (discrete-time points at weeks 4, 8, and 20, treating week 0 as the reference) and treatment by time interaction^48^. The baseline score of the same domain was included as a covariate. This parameterization allowed the use of statistical contrasts to evaluate specific hypotheses, for example, whether or not acupuncture treatment yielded a greater BPI pain reduction than CBT-I from baseline to week 8.

We also dichotomized each study participant into a responder or non-responder by insomnia outcome and by pain outcome. We defined insomnia responders as those with a reduction ≥8 in ISI total scores from week 0 to week 8 or those who achieved an ISI score <8 after treatment. We defined pain responders as those with a reduction of 33% or greater in BPI severity scores from week 0 to week 8. Next, we performed responder analyses, in which we evaluated the difference in pain reduction between insomnia responders and non-responders, and the difference in insomnia outcomes between pain responders and non-responders. We also conducted linear mixed-effects models on pain severity scores at weeks 4, 8, and 20 as a function of responder status (insomnia responder or non-responder), time, and time by responder status interaction. Baseline scores of the same domain were entered as a covariate.

The sample size was predetermined by the original study according to the primary outcome of insomnia severity^49^. Statistical hypothesis testing was based on the two-sided alpha-error rate of 0.05. The goal of the analyses performed in this study was hypothesis-generating rather than confirmatory. All statistical analyses were conducted using Stata (version 15.0; StataCorp LLC, College Station, Texas) and SAS (version 9.4; SAS Institute Inc., Cary, North Carolina) software.

### Reporting summary

Further information on research design is available in the Nature Research Reporting Summary linked to this article.

## Supplementary information

Supplementary Material

Reporting Summary

## Data Availability

The data that support the findings of this study are not openly available due to human data and are available from the corresponding author (Jun J. Mao: maoj@mskcc.org) upon reasonable request.

## Code availability

The code generated during the statistical analysis is available from the corresponding author (Jun J. Mao: maoj@mskcc.org) upon reasonable request. We generated the code using Stata (version 15.0; StataCorp LLC, College Station, Texas) and SAS (version 9.4; SAS Institute Inc, Cary, North Carolina) software.
