# Supplementary material for: Acupuncture versus cognitive behavioral therapy for pain among cancer survivors with insomnia: an exploratory analysis of a randomized clinical trial
Source: NPJ Breast Cancer. 2021 Nov 30;7:148. doi: 10.1038/s41523-021-00355-0 (PMC8633385; doi:10.1038/s41523-021-00355-0)
Supplement: Supplementary file 1 — Supplementary Material [file 41523_2021_355_MOESM1_ESM.pdf]

SUPPLEMENTARY MATERIAL

Acupuncture Protocol for Insomnia

The following protocol was developed based on Traditional Chinese Medicine Acupuncture Textbook (1) with incorporation of points that treat pain, anxiety, and fatigue, which are common in cancer survivors (2,3). The protocol was developed in consultation with local acupuncturists and acupuncturists in China.

This protocol served as a guide for the acupuncturist(s) to administer the acupuncture procedure:

Assessment:

1) History
2) Tongue diagnosis
3) Pulse diagnosis

Points Selection:

1) Core points for insomnia:
   Bilateral: Shenmen (HT7), Sanyinjiao (SP6)
   Midline: Baihui (GV20), Shenting (GV24)
   Unilateral: Shenmen (Auricular), Sympathetic (Auricular)
2) As focal pain, generalized aches, anxiety, and fatigue are common in cancer survivors, use Table 1 to pick at least 2 supplemental points
3) Table 1 is meant to serve as a guide, but if the acupuncturist wishes to use an additional point not described in Table 1 based on their clinical judgment for that specific patient case, additional points can be used, but must be sufficiently documented.
4) Limit the total of points to 8-16

Needle Type:

Seirin Needles: (Manufactured in Japan, Distributed by Lhasa OMS, Weymouth, MA)

1) Auricular Points: 0.16mm x 15mm-30mm
2) Body Points: 0.16mm-0.25mm x 30mm-40mm

Acupuncture Administration:

1) Assist patient to lie comfortably on a table
2) Wipe the needling points with alcohol
3) Insert needle to appropriate depth with brief stimulation to achieve “De Qi” sensation
4) Set timer for 30 minutes
5) Document acupuncture procedure
6) Check patient in 15 minutes for comfort
7) Remove needles and wipe any blood with a gauze or cotton tipped applicator

8) Assist patient to slowly get up from the examination table
9) Complete documentation using the case report form

Training of acupuncturist:

Four licensed acupuncturists with 11–14 years of experience delivered the interventions. They received training on the specific protocol, completed study checklists to ensure treatment fidelity, and J.J.M. checked documentation and provided feedback.

References

1) Cheng X, editor. Chinese acupuncture and moxibustion. Beijing, China: Foreign Languages Press; 1987.
2) Mao JJ, Bruner DW, Stricker C, Farrar JT, Xie SX, Bowman MA, et al. Feasibility trial of electroacupuncture for aromatase inhibitor-related arthralgia in breast cancer survivors. Integr Cancer Ther. 2009 Jun;8(2):123-9.
3) Mao JJ, Xie SX, Farrar JT, Stricker CT, Bowman MA, Bruner D, et al. A randomised trial of electro-acupuncture for arthralgia related to aromatase inhibitor use. Eur J Cancer. 2014 Jan;50(2):267-76.

Supplementary Table 1: Acupuncture Point Se lection for Supplemental Points

**Symptoms**	**Acupuncture Points (Please indicate by circling)**					
Focal Pain Locations						
Shoulder	Jianyu (L.I.15)	Jianliao (S.J.14)	Jianzhen (S.I.9)	Naoshu (S.I.10)	Jianneiling (Ext ra)	--
Scapula	Tianzong (S.I.11)	Bingfeng (S.I.12)	Jianwaishu (S.I.14)	Gaohuangshu (U.B.43)		--
	Quchi (L.I.11)	Chize (Lu. 5)	Tianjing (S.J.10)	Waiguan (S.J.5)	Hegu(L.I.4)	--
Hand/	Houxi (S.I.3)	Sanjian (L.I.3)	Baxie (Extra)	Hegu (L.I.4)	--	--
Finger hip	Huantiao (G.B.30)	Yinmen (U.B.37)	Juliao (G.B.29)	--	--	--
Knee	Lianqiu (St.34)	Dubi (St. 35)	Nei Xiyan (Ext ra)	Yanlingquan (G.B.34)	Xiangguan (G.B. 35)	Yinlingquan (Sp. 9)
Leg	Chengshan (U.B.57)	Feiyang (U.B.58)	--	--	--	--
Ankle	Jiexi (St.41)	Shangqui (Sp. 5)	Quixu (G.B.40)	Kunlun (U.B.60)	Taixi (K.3)	--
Foot/	Gongsun (Sp.4)	Shugu (U.B.65)	Bafeng (Ext ra)	Taichong (Liv.3)	--	--
Toe Neck	Tianzhu (U.B.10)	Fengchi (G.B.20)	Bailao (Ext ra)	Jianjing (G.B.21)	--	--
Low Back	Shenshu (U.B.23)	Dachangshu (U.B.25)	Yaoyan (Ext ra)	Pigen (Ext ra)	Weizhong (U.B.40)	--
General symptoms						
Psychological distress	Neiguan (P.C. 6)	Taichong (Liv. 3)	--	--	--	--
Generalized fatigue	Qihai (CV. 6)	Zusanli (St.36)	--	--	--	--
