# Supplementary material for: Acupuncture versus cognitive behavioral therapy for pain among cancer survivors with insomnia: an exploratory analysis of a randomized clinical trial
Source: NPJ Breast Cancer. 2021 Nov 30;7:148. doi: 10.1038/s41523-021-00355-0 (PMC8633385; doi:10.1038/s41523-021-00355-0)
Supplement: Supplementary file 2 — Reporting Summary [file 41523_2021_355_MOESM2_ESM.pdf]

nature portfolio

Corresponding author(s): Jun J. Mao
Mingxiao Yang

Last updated by author(s): Oct 14, 2021

Reporting Summary

Nature Portfolio wishes to improve the reproducibility of the work that we publish. This form provides structure for consistency and transparency in reporting. For further information on Nature Portfolio policies, see our Editorial Policies and the Editorial Policy Checklist.

Statistics

For all statistical analyses, confirm that the following items are present in the figure legend, table legend, main text, or Methods section.

n/a	Confirmed	
☐	☒	The exact sample size (n) for each experimental group/condition, given as a discrete number and unit of measurement
☐	☒	A statement on whether measurements were taken from distinct samples or whether the same sample was measured repeatedly
☐	☒	The statistical test(s) used AND whether they are one- or two-sided<br>*Only common tests should be described solely by name; describe more complex techniques in the Methods section.*
☐	☒	A description of all covariates tested
☐	☒	A description of any assumptions or corrections, such as tests of normality and adjustment for multiple comparisons
☐	☒	A full description of the statistical parameters including central tendency (e.g. means) or other basic estimates (e.g. regression coefficient) AND variation (e.g. standard deviation) or associated estimates of uncertainty (e.g. confidence intervals)
☐	☒	For null hypothesis testing, the test statistic (e.g. F, t, r) with confidence intervals, effect sizes, degrees of freedom and P value noted<br>*Give P values as exact values whenever suitable.*
☒	☐	For Bayesian analysis, information on the choice of priors and Markov chain Monte Carlo settings
☒	☐	For hierarchical and complex designs, identification of the appropriate level for tests and full reporting of outcomes
☒	☐	Estimates of effect sizes (e.g. Cohen's d, Pearson's r), indicating how they were calculated

Our web collection on statistics for biologists contains articles on many of the points above.

Software and code

Policy information about availability of computer code

Data collection	Study data were collected and managed using REDCap electronic data capture tools hosted at MSKCC. REDCap (Research Electronic Data Capture) is a secure, web-based software platform designed to support data capture for research studies, providing 1) an intuitive interface for validated data capture; 2) audit trails for tracking data manipulation and export procedures; 3) automated export procedures for seamless data downloads to common statistical packages; and 4) procedures for data integration and interoperability with external sources.
Data analysis	All statistical analyses were conducted using Stata (version 15.0; StataCorp LLC, College Station, Texas) and SAS (version 9.4; SAS Institute Inc, Cary, North Carolina) software.

For manuscripts utilizing custom algorithms or software that are central to the research but not yet described in published literature, software must be made available to editors and reviewers. We strongly encourage code deposition in a community repository (e.g. GitHub). See the Nature Portfolio guidelines for submitting code & software for further information.

Data

Policy information about availability of data

All manuscripts must include a data availability statement. This statement should provide the following information, where applicable:

- Accession codes, unique identifiers, or web links for publicly available datasets
- A description of any restrictions on data availability
- For clinical datasets or third party data, please ensure that the statement adheres to our policy

The data that support the findings of this study are not openly available due to human data and are available from the corresponding author (Jun J. Mao: maoj@mskcc.org) upon reasonable request.

Field-specific reporting

Please select the one below that is the best fit for your research. If you are not sure, read the appropriate sections before making your selection.

☒ Life sciences ☐ Behavioural & social sciences ☐ Ecological, evolutionary & environmental sciences

For a reference copy of the document with all sections, see nature.com/documents/nr-reporting-summary-flat.pdf

Life sciences study design

All studies must disclose on these points even when the disclosure is negative.

Sample size	The sample size was predetermined by the original study according to the primary outcome of insomnia severity .
Data exclusions	No data excluded
Replication	Not applicable as this study is a clinical trial.
Randomization	A permuted block randomization mechanism
Blinding	Study investigators (PI, co-investigators, and statisticians) were masked to group assignment. Patients, research staff, and treatment therapists were not masked.

Reporting for specific materials, systems and methods

We require information from authors about some types of materials, experimental systems and methods used in many studies. Here, indicate whether each material, system or method listed is relevant to your study. If you are not sure if a list item applies to your research, read the appropriate section before selecting a response.

Materials & experimental systems

n/a	Involved in the study	
☒	☐	Antibodies
☒	☐	Eukaryotic cell lines
☒	☐	Palaeontology and archaeology
☒	☐	Animals and other organisms
☐	☒	Human research participants
☐	☒	Clinical data
☒	☐	Dual use research of concern

Methods

n/a	Involved in the study	
☒	☐	ChIP-seq
☒	☐	Flow cytometry
☒	☐	MRI-based neuroimaging

Human research participants

Policy information about studies involving human research participants

Population characteristics	Of the160 participants randomized to receive acupuncture (n=80) or CBT-I (n=80), 35 in each group had a baseline worst pain value of 4 or greater and were included in this analysis (Figure 1). Of those assigned to acupuncture, 31 completed the required treatment and 32 completed the 20-week follow-up. Of those assigned to CBT-I, 30 completed the study interventions and 29 completed the 20-week follow-up. All 70 participants were included in ITT analyses. The demographics and clinical characteristics are detailed in Table 1.
Recruitment	After initial screening, trained research staff interviewed interested participants to confirm an insomnia diagnosis and additional inclusion/exclusion requirements. Confirmed eligible patients were scheduled to complete baseline sleep assessment after providing written informed consent.
Ethics oversight	The original study was completed from March 2015 to July 2017. The institutional review boards at the University of Pennsylvania and Memorial Sloan Kettering Cancer Center approved the study procedures summarized below.

Note that full information on the approval of the study protocol must also be provided in the manuscript.

Clinical data

Policy information about clinical studies

All manuscripts should comply with the ICMJE guidelines for publication of clinical research and a completed CONSORT checklist must be included with all submissions.

Clinical trial registration	ClinicalTrials.gov Identifier NCT02356575, registration date Feb 5, 2015

Study protocol	[39] Garland SN, Gehrman P, Barg FK, Xie SX, Mao JJ. CHoosing Options for Insomnia in Cancer Effectively (CHOICE): Design of a patient centered comparative effectiveness trial of acupuncture and cognitive behavior therapy for insomnia. Contemp Clin Trials. 2016;47:349-55.
Data collection	University of Pennsylvania and Memorial Sloan Kettering Cancer Center
Outcomes	The BPI is an 11-item pain assessment tool validated for use with cancer patients [32]. It measures the intensity of pain (four items) and interference (seven items) of pain in the patient's life. The average score on the four pain severity items is the primary outcome for this analysis. The BPI psychometrics are well-established (Cronbach's alpha 0.80-0.87 for the four pain severity items and 0.89-0.92 for the seven interference items). Participants who reported a reduction of 33% or greater on average pain intensity after treatment were considered pain responders [33].<br>The ISI is a validated patient-reported outcome of insomnia designed to specifically assess the impact of insomnia on daytime functioning and the severity of associated distress [34]. The ISI includes seven items that are scored on a five-point scale ranging from 0 to 4 with higher scores representing more severe insomnia symptoms. The optimal cutoff scores are 0-7 (no clinically significant sleep difficulties), 8-14 (sleep difficulties warrant further investigation), and 15+ (presence of clinically significant insomnia). The ISI has demonstrated internal consistency, reliability, construct validity, specificity, and sensitivity [35]. Participants who report a ≥8 reduction in ISI score or achieve an ISI score <8 after treatment are considered insomnia responders [36, 37].
